# Heterologous expression of human costimulatory molecule B7-2 and construction of B7-2 immobilized polyhydroxyalkanoate nanoparticles for use as an immune activation agent

**DOI:** 10.1186/1472-6750-12-43

**Published:** 2012-07-30

**Authors:** Ming-Chuan Li, Qian-Qian Liu, Xiao-Yun Lu, Ya-Li Zhang, Lei-Lei Wang

**Affiliations:** 1 Department of Biological Science and Bioengineering, Key Laboratory of Biomedical Information Engineering of Ministry of Education, School of Life Science and Technology, Xi’an Jiaotong University, Xi’an 710049, Shaanxi, P. R. China

**Keywords:** PHA nanoparticle, B7-2, Costimulation, PhaP, Immobilization

## Abstract

**Background:**

Costimulation of T cells via costimulatory molecules such as B7 is important for eliciting cell-mediated antitumor immunity. Presenting costimulation molecules by immobilizing recombinant B7 on the surface of nanovectors is a novel strategy for complementary therapy. Polyhydroxyalkanoates (PHAs) are a family of biodegradable, non-toxic, biocompatible polyesters, which can be used as a nonspecific immobilizing matrix for protein presentation. Recombinant protein fusion with PHA granule binding protein phasin (PhaP) can be easily immobilized on the surface of PHA nanoparticles through hydrophobic interactions between PhaP and PHA, and therefore provides a low-cost protein presenting strategy.

**Results:**

In this study, the extracellular domain of the B7-2 molecule (also named as CD86) was fused with PhaP at its N-terminal and heterogeneously expressed in recombinant *Escherichia coli* strain BL21 (DE3). The purified B7-2-PhaP protein was immobilized on the surface of poly(3-hydroxybutyrate-co-3-hydroxyhexanoate) (PHBHHx)-based nanoparticles. Loading of 240 μg (3.2 pMol) of B7-2-PhaP protein per mg nanoparticles was achieved. Immobilized B7-2-PhaP on PHBHHx nanoparticles induced T cell activation and proliferation *in vitro*.

**Conclusions:**

A PHA nanoparticle-based B7-2 costimulation molecule-presenting system was constructed. The PHA-based B7 presenting nanosystem provided costimulation signals to induce T cell activation and expansion *in vitro*. The B7-2-PhaP immobilized PHA nanosystem is a novel strategy for costimulation molecule presentation and may be used for costimulatory molecule complementary therapy.

## Background

It is generally accepted that the activation of naïve T cells requires two signals. One is provided by association of the T-cell receptor with the major histocompatibility complex–peptide complex. The other, known as costimulation, is necessary for optimal T-cell activation, which leads to clonal T-cell expansion, cytokine secretion and development of effector functions. Two main costimulatory molecules, B7-1 and B7-2, accomplish the coactivation function through binding with their ligand CD28 on the surface of T cells [1]. Lack of costimulation however, results in the induction of T-cell tolerance, named anergy. This is important for tumor immunity because a variety of tumors are potentially immunogenic but do not stimulate an effective anti-tumor immune response *in vivo*. That is partly because some tumors can deliver antigen-specific signals to T cells, but do not deliver costimulatory signals necessary for full activation of T cells [2].

Low levels of B7-1 and B7-2 have been detected on certain human cancers [3,4] and in a series of cell lines derived from human carcinomas [2,5]. Absence of costimulatory B7 molecule expression renders tumors “invisible” to the immune system. Accordingly, expression of B7 on tumor cells induced tumor rejection in a murine model, suggesting that providing extra costimulation molecules might render tumor cells capable of effective antigen presentation, leading to their eradication *in vivo*. Based on the safety considerations of expressing B7 using a virus vector, we propose a novel strategy to present costimulatory molecules by immobilizing recombinant B7 on the surface of polyhydroxyalkanoate (PHAs)-based nanoparticles.

PHAs are a family of biodegradable, non-toxic, biocompatibility polyesters, which are synthesized by a wide range of microorganisms [6,7], have promise as biomedical materials and have been investigated for many transplant applications [8]. Moreover, PHAs could also be used as a nonspecific immobilizing matrix for protein purification and presentation, mediated by several kinds of PHA granule associated proteins, which contain a hydrophobic granule-binding domain [9-13]. Among these proteins, PHA granule binding protein phasin (PhaP) has a high affinity for hydrophobic materials and has been utilized to develop a low-cost protein production system [11,12], as well as a fluorescence activated cell sorting-based diagnostic technique [13].

In this study, the extracellular domain of the B7-2 molecule was fused with PhaP at its N-terminal and the recombinant B7-2-PhaP protein was immobilized on the surface of poly(3-hydroxybutyrate-*co*-3-hydroxyhexanoate) (PHBHHx)-based nanoparticles. The immunocompetence of the resulting fusion protein and functionality of the PHBHHx nanoparticles were analyzed.

## Results

### Microbial production and purification of GST-B7-2-PhaP fusion proteins by *Escherichia coli*

**Figure 1:**
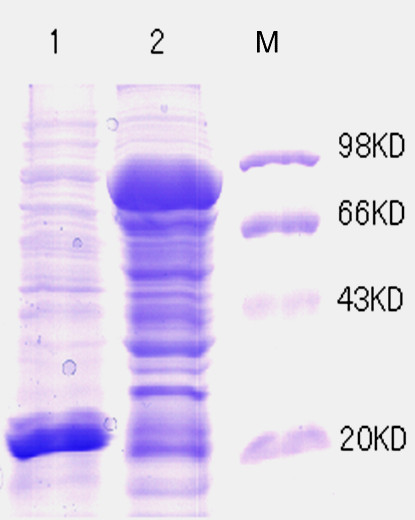
**Coomassie blue-stained SDS-PAGE of hetero-expressed GST-B7-2-PhaP fusion protein by recombinant *E. coli *BL21 (DE3) (pGEX-B72P).** Lane 1: whole crude lysate of *E. coli* BL21 (DE3) transformed with pGEX-4T-3 plasmid. Lane 2: whole crude lysate of *E. coli* BL21 (DE3) transformed with pGEX-B72P plasmid. Protein weight marker (Institute of Biochemistry and Cell Biology, SIBS, CAS, China) was loaded in lane M.

**Figure 2:**
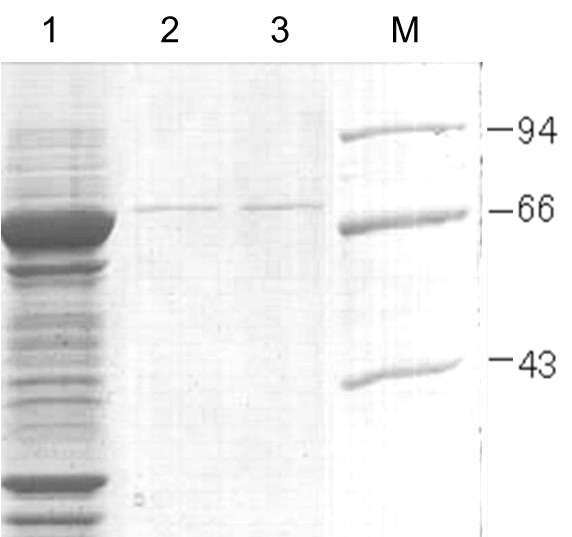
**Coomassie blue-stained SDS-PAGE of soluble GST-B7-2-PhaP fusion protein.** Lane 1: the soluble fraction was recovered after centrifugation of whole crude lysate of *E. coli* BL21 (DE3) transformed with pGEX-B72P plasmid. Lane 2: purified soluble GST-B7-2-PhaP fusion protein. Protein weight marker (Institute of Biochemistry and Cell Biology, SIBS, CAS, China) was loaded in lane M.

The PhaP gene was fused with the coding sequence for the extracellular domain of the B7-2 molecule and was expressed in *Escherichia coli* BL21 (DE3), with a glutathione S-transferase (GST) tag at the N-terminal of the resulting fusion protein. A flexible peptide linker (GGGGSGGGSGGGS) was inserted between B7-2 and PhaP to ensure correct folding. The GST-B7-2-PhaP fusion protein was efficiently produced by recombinant *E. coli* BL21 (DE3) (Figure 1). However, most of the expressed intracellular fusion protein formed inclusion bodies, probably owing to the hydrophobic nature of PhaP. In order to increase the soluble proportion of the fusion protein to facilitate purification, 5% (v/v) ethanol was added to the broth during cultivation. At the same time, the process of inducing expression was undertaken at a low temperature of 20°C. The soluble crude proteins obtained from lysates of *E. coli* were collected by sonication and centrifugation. The GST-B7-2-PhaP fusion protein was purified by affinity chromatography using a glutathione sepharose 4B (GS4B) column. Sodium dodecyl sulfate (SDS) polyacrylamide gel electrophoresis (PAGE) analysis showed that soluble GST-B7-2-PhaP fusion protein with a molecular mass of 73 kDa was successfully produced in the recombinant *E. coli* BL21 (DE3) and a single band of the purified GST-B7-2-PhaP fusion protein was detected from the eluted sample (Figure 2).

### Preparation and characterization of nanoparticles

**Figure 3:**
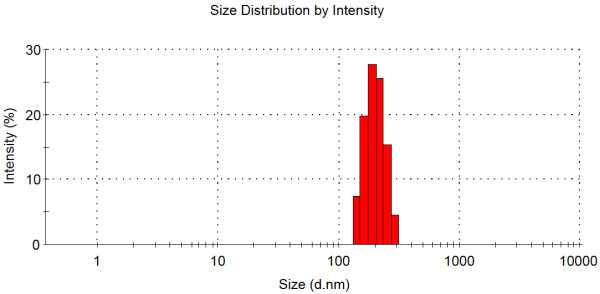
**Size distribution of PHBHHx nanoparticles determined by dynamic light scattering**.

PHBHHx nanoparticles were prepared using the emulsification–solvent evaporation technique described previously [9,14]. The average diameter of prepared nanoparticles was 198 ± 5 nm with a relative low polydispersity index of 0.009. Figure 3 shows the size distribution of resulting PHBHHx nanoparticles, which formed an incipient stable colloidal suspension in aqueous medium that could be stored for at least 1 month without aggregation. The concentration of the PHBHHx nanoparticle suspension was adjusted to 5 mg ml^-1^ for further application.

### Characterization of GST-B7-2-PhaP fusion protein immobilized nanoparticles

**Figure 4:**
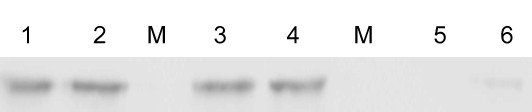
**Western blot analysis of GST-B7-2-PhaP fusion protein immobilized on the surface of PHBHHx nanoparticles.** Lanes 1-2: the original GST-B7-2-PhaP protein samples with an initial concentration of 2 and 2.5 mg ml^-1^, respectively. Lanes 3-4: GST-B7-2-PhaP protein eluted from the surface of PHBHHx nanoparticles after the immobilization procedure. Lanes 5-6: the remnant GST-B7-2-PhaP proteins remaining in the supernatant after the immobilization procedure. The concentration of the initial protein sample of Lanes 3 and 5 was 2 mg ml^-1^, and that of Lanes 4 and 6 was 2.5 mg ml^-1^. Protein weight marker (Institute of Biochemistry and Cell Biology, SIBS, CAS, China) was loaded in lane M, which was not detected by western blot analysis.

To characterize the nanoparticles, 250 μl of 5 mg ml^-1^ PHBHHx nanoparticles were incubated with an equal volume of purified GST-B7-2-PhaP fusion proteins (1-2.5 mg ml^-1^) for 16 h and then collected by centrifugation. After the immobilization process and removal of the nanoparticle proportion, no proteins were detected in the supernatant of samples with the initiation GST-B7-2-PhaP concentration of 1, 1.5 and 2 mg ml^-1^. In the sample to which 2.5 mg ml^-1^ GST-B7-2-PhaP protein was added, 0.236 mg ml^-1^ protein remained in the supernatant after removing the coated nanoparticles. This indicated that approximately 400 μg of GST-B7-2-PhaP fusion protein could be immobilized on the surface of 1 mg PHBHHx nanoparticles. Western blot analysis also demonstrated that GST-B7-2-PhaP fusion proteins could be detected on the samples eluted from the nanoparticles (Figure 4, Lane 3, 4). A slight amount of protein could still be detected in the supernatant when the initial protein concentration was 2.5 mg ml^-1^ (Figure 4, Lane 6). However, when the protein concentration of the initial sample was 2 mg ml^-1^, all GST-B7-2-PhaP fusion proteins could be immobilized on the surface of PHBHHx nanoparticles and no protein was detected in the supernatant after incubation with PHBHHx nanoparticles (Figure 4, Lane 5). This demonstrated that the highest achievable amount of immobilized GST-B7-2-PhaP fusion protein on the PHBHHx nanoparticles could be as high as 400 μg per 1 mg nanoparticles.

### Proliferation of human T cells stimulated with B7-2-PhaP-PHBHHx nanoparticles

**Figure 5:**
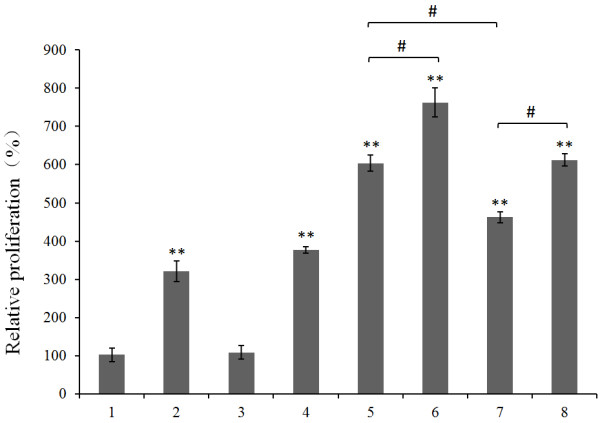
**Relative proliferation ratio of human peripheral blood lymphocytes by B7-2-PhaP immobilized PHBHHx nanoparticle treatment.** Lymphocytes were seeded in triplicate at 1 × 10^4^ cells per well in 96-well culture plates, followed by incubation with the indicated treatments for 72 h. 1: control cells; 2: cells treated with 5 μg ml^-1^ phytohemagglutinin; 3: cells treated with 500 ng ml^-1^ anti-CD3 antibody; 4: cells treated with 5 μg ml^-1^ PHBHHx nanoparticles; 5: cells treated with 1 μg ml^-1^ B7-2-PhaP fusion protein loaded PHBHHx nanoparticles; 6: cells treated with 1 μg ml^-1^ B7-2-PhaP fusion protein loaded PHBHHx nanoparticles plus 500 ng ml^-1^ anti-CD3 antibody; 7: cells treated with 1 μg ml^-1^ non-immobilized B7-2-PhaP fusion protein; 8: cells treated with 1 μg ml^-1^ non-immobilized B7-2-PhaP fusion protein plus 500 ng ml^-1^ anti-CD3 antibody. The cell proliferation was analyzed by MTT assay and the relative proliferation ratio was calculated relative to the cell proliferation level of the control group, which was defined as 100%.

Human lymphocytes were isolated from peripheral blood donated by a healthy volunteer. The T cell population was expanded by phytohemagglutinin stimulation. The assembled GST-B7-2-PhaP-PHBHHx nanoparticles were digested with thrombin to remove the GST tag. The immunocompetence of B7-2-PhaP-PHBHHx nanoparticles was evaluated by T cell proliferation assay. Figure 5 shows the relative proliferation of human T cells under different treatments.

Following *in vitro* phytohemagglutinin (5 μg ml^-1^) stimulation, activated T cells proliferated. T cells treated with anti-CD3 antibody (5 ng ml^-1^) alone received only the primary signal for T cell activation, and thus did not become activated or proliferate. However, addition of either immobilized B7-2-PhaP (1 μg ml^-1^) or free B7-2-PhaP protein led to significant cell expansion. The B7-2-PhaP loaded PHBHHx nanoparticles showed a higher activity for stimulated cell expansion than non-immobilized free B7-2-PhaP protein. PHBHHx nanoparticle treatment also induced the stimulation of cell proliferation. Immobilized B7-2-PhaP on PHBHHx nanoparticles together with anti-CD3 antibody demonstrated the highest activity for the stimulation of cell proliferation. These results indicated that PHBHHx-based B7-2-PhaP presenting nanoparticles can be used to activate T cells.

## Discussion

PHAs are linear biopolyesters produced as energy- and carbon-storage materials by many bacteria. The medical applications of PHA have been extensively explored in recent years for implant biomedical applications including sutures, nerve conduits, patches, slings, cardiovascular patches, stents, guided tissue repair/regeneration devices, bone marrow scaffolds and so on. PHA nanoparticles have also been developed as controlled drug-release vectors owing to their biocompatibility and biodegradability.

The surface of PHA-based nanoparticles can be modified by many different types of signaling molecules and thus can be used for purposes other than targeted delivery. This can also be achieved by engineering proteins other than PhaP such as other PHA surface associated proteins including PhaC, PhaR, and PhaZ. These proteins provide targets for displaying various molecules on polymer nanoparticle surfaces for protein immobilization and display. The phasin PhaP is a small amphiphilic protein that can associate with most hydrophobic polymer granule surfaces via strong hydrophobic interactions. PhaP is abundant on the surface of natural PHA granules, and can reach approximately 5% (wt/wt) of the total cell protein in PHA-accumulating bacteria. Thus, multifunctional polymer nanoparticles can be obtained through protein engineering of PhaP and applied to a variety of versatile situations. The functional fusion proteins were able to produce easily by recombinant bacteria cultivation. This method provides a simple and convenient way to immobilize and present proteins on the surface of hydrophobic biomaterials without complicated procedures to modify both proteins and materials.

Human B7-2 is a 329-amino-acid (aa) protein containing a putative 23-aa signal peptide, a 224-aa extracellular domain, a 21-aa transmembrane domain, and a 61-aa cytoplasmic domain. The absence of the signal peptide, the transmembrane domain, and the cytoplasmic domain did not affect the costimulatory activity of B7 molecules. In this study, the 224-aa extracellular domain was subcloned and fused with PhaP and immobilized on the surface of PHBHHx nanoparticles through the hydrophobic interactions between PhaP and polymer. As measured by western blot, 400 μg of GST-B7-2-PhaP fusion proteins could be immobilized on the surface of 1 mg PHBHHx nanoparticles, which by extrapolation implies 240 μg (3.2 pMol) B7-2-PhaP fusion protein was present on the surface of 1 mg of nanoparticles. When 1 μg ml^-1^ immobilized B7-2-PhaP proteins was applied to cultured cells, the amount of nanoparticles used was approximately 4.2 μg ml^-1^. Previously we demonstrated that 5 μg ml^-1^ PHBHHx nanoparticles could enhance the cell viability of rodent lymphocytes from spleen. Interestingly, the main degradation product of the PHBHHx polymer, 3-hydroxybutyrate, can promote interleukin-2 and interferon-γ secretion by rodent T lymphocytes (data not published). This may explain the slight T cell proliferation resulting from the non-coated PHBHHx nanoparticle treatment in this study.

Phytohemagglutinin is a lectin found in plants, and is used as a mitogen to trigger T lymphocyte cell division. Isolated lymphocytes were treated with 5 μg ml^-1^ phytohemagglutinin to increase cell numbers prior to the proliferation assay. Thus, the expanded cells were mainly T cells, although small numbers of B cells and other monocytes remained in the culture. This may explain why non-immobilized B7-2-PhaP fusion proteins also resulted in cell proliferation. PhaP is a bacterial protein that might be recognized by B cells, thus triggering an immune response resulting in lymphocyte proliferation. However, whether the B7-2-PhaP fusion protein was immobilized or not, the combined treatment of B7-2-PhaP protein and anti-CD3 antibody achieved the highest proliferation ratio and number of activated T cells. Compared with the non-immobilized free B7-2-PhaP protein, the B7-2-PhaP protein loaded nanoparticles showed a higher activity for cell proliferation stimulation. This can be explained by the advantage of the immobilization system, which can be used to provide several signal molecules that interact concurrently with ligands on cell surfaces.

## Conclusions

In this study, a novel PHA nanoparticle-based B7 costimulation molecule-presenting system was developed. Both the vehicle and the presented functional protein were produced by microorganism fermentation and purified from the culture broth. The extracellular domain of the B7-2 molecule was fused with PhaP, and the B7-2-PhaP protein was hetero-expressed by recombinant *E. coli* BL21 (DE3). The purified B7-2-PhaP protein was immobilized on the surface of PHBHHx nanoparticles through the hydrophobic interaction between PhaP and polymer. The immunocompetence study showed that the PHA-based B7 presenting nanosystem could provide costimulatory signals for T cell activation, leading to T cell expansion *in vitro*. The B7-2-PhaP immobilized PHA nanosystem provides a novel strategy for the presentation of costimulatory molecules and may be a useful tool for costimulation molecule complementary therapy.

## Methods

### Plasmid construction

**Figure 6:**
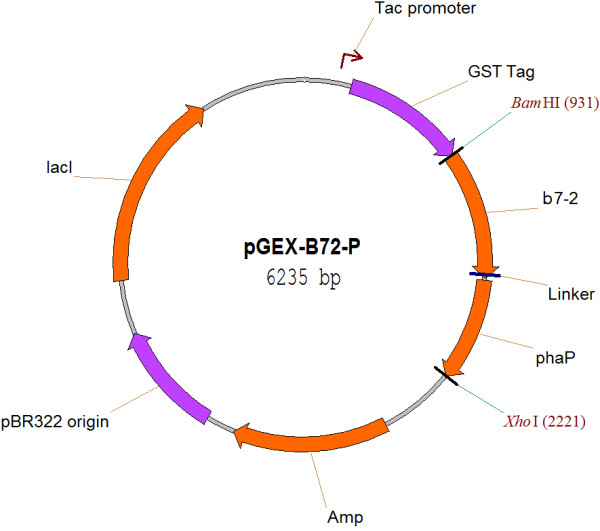
**Plasmid map of pGEX-B72-P.** pGEX-B72-P was derived from plasmid pGEX-4T-3 which contains the pBR322 origin. B7-2: coding sequence for 224-aa extracellular domain of human B7-2 costimulatory molecule; phaP: coding sequence of PHA associated protein phasin (PhaP); Amp: ampicillin resistance gene; Lac I: coding sequence of Lac operon DNA-binding transcriptional repressor.

The DNA sequence coding for the extracellular domain of human B7-2 molecule (52-672bp) was subcloned from the plasmid B7-2(IgV + C)/TEasy, which contains the cDNA sequence of B7-2. The gene encoding PhaP of *Ralstonia eutropha* H16 was subcloned from the plasmid pTwin2-EPhaP (donated by the Laboratory of Microbiology, Department of Biological Science and Biotechnology, Tsinghua University, Beijing, China). The primers B1 (5’-GCGGATCCATGGCTCCTCTGAAGATTC-3’) (the double line marks the *Bam*HI restriction site) and B2 (5’-ACCAGAGCCACCTCCTGAACCGCCTCCACCTGTAATCCAAGGAATG-3’) were used to amplify extracellular domain of B7-2 coding sequence. The primers P1 (5’-GGTTCAGGAGGTGGCTCTGGTGGATCGCTCACCCCGGAACAAGTT-3’) and P2 (5’-CGCTCGAGAGCCGTCGTCTTCTTTGCCGT-3’) (the double line marks the *Xho*I restriction site) were used to amplify the PhaP gene. The PCR products of B7-2 and PhaP were mixed at the ratio of 1:1 and fusion with each other by overlap extension PCR. Then *BamH*I and *Xho*I digested B7-2-*phaP* segment was inserted into the corresponding sites downstream of the GST tag coding sequence of pGEX-4T-3 expression vector to generate the final plasmid pGEX-B72P (Figure 6).

### Cultivation of strains and expression of recombinant protein

*E. coli* strain BL21 (DE3) was used to express GST-B7-2-PhaP fusion proteins. *E. coli* BL21 (DE3) transformants carrying the pGEX-B72P plasmid was cultured at 37°C in 500 ml shake flasks containing 200 ml Luria Bertani medium (1% w/v Bacto tryptone, 0.5% yeast extract, and 1% NaCl) supplemented with 100 mg ml^-1^ ampicillin and 3% (v/v) ethanol in a shaking incubator at 200 rpm. At an OD_600_ of 0.4-0.6, 0.1 mM isopropyl β-D-thiogalactopyranoside was added to induce the expression of the fusion gene and the recombinant *E. coli* BL21 (DE3) carrying pGEX-B72P was continued in culture at 20°C for another 6 hours.

### Recombinant protein purification and characterization

Cells were harvested by centrifugation at 6000 × *g* for 10 min and re-suspended in 20 ml phosphate-buffered saline (PBS) buffer followed by sonication (Ultrasonic crasher, Scientz-II D, Ningbo, China) for 10 min at 40% output. After centrifugation at 12000 × *g* for 30 min at 4°C, the clarified soluble protein fraction was collected. The target recombinant GST-B7-2-PhaP fusion protein was purified by GS4B affinity chromatography (GE Healthcare, USA) according to the manufacturer’s protocol.

The eluted protein was purified and condensed by the dialysis bag method. Sample collected after purification was analyzed by 12% SDS-PAGE. The purified recombinant protein was stored at −20°C after lyophilization for further usage.

### Preparation of PHBHHx nanoparticles

The PHBHHx material was produced, isolated and characterized as described previously [15,16]. The PHBHHx nanoparticles were fabricated by a modified emulsification/solvent diffusion method [17,18]. Briefly, 20 mg of PHBHHx polymer was added into 1 ml chloroform. Five milliliters of 1% poly(vinyl alcohol) (PVA) (w/v) was sonicated for 1 min and was slowly added to 1 ml of organic solution. The double emulsion was then sonicated using a probe sonicator (Sonics & Materials, Newtown, CT, USA) for 5 min and the mixed solution was moderately stirred with a magnetic mixer for 6 h to solidify the nanodroplets. Chloroform was removed by volatilization at room temperature. The nanoparticles were collected by centrifugation at 38000 rpm for 60 min, followed by washing twice with deionized water. The particle size was analyzed by a laser light scattering machine (Zetasizer Nano ZS, Malvern, UK).

### Immobilization and characterization of fusion proteins on nanoparticles

Fusion protein GST-B7-2-PhaP was dissolved in PBS at a final concentration of 1-2.5 mg ml^-1^. A concentration of 5 mg ml^-1^ of PHBHHx nanoparticles was mixed with various amounts of purified GST-B7-2-PhaP fusion proteins, followed by stirring and incubation at 4°C for 12-16 h. Subsequently, the colloidal system was collected by centrifugation at 12,000 × *g* for 30 min and washed twice with PBS. The immobilized GST-B7-2-PhaP fusion protein was calculated according to the difference between the amounts of protein in the initial sample and that in the supernatant after incubation, and was quantified using the bicinchoninic acid method using a protein assay kit Beyotime (#P0010, Beyotime Institute of Biotechnology, China) according to the manufacturer’s protocol.

The assembled GST-B7-2-PhaP-PHBHHx nanoparticles were resuspended in protein loading buffer (50 mM Tris-HCl (pH 6.8), 2% SDS, 0.1% bromophenol blue, 10% glycerol, 1% β-mercaptoethanol) and boiled for 2 min to allow detachment of denatured GST-B7-2-PhaP fusion protein from PHBHHx nanoparticles. After 12,000 × *g* centrifugation for 10 min, the supernatant was analyzed by SDS-PAGE and western blot to verify the immobilization of GST-B7-2-PhaP on the surface of PHBHHx nanoparticles. Briefly, proteins were transferred to poly (vinylidene fluoride) (PVDF) membranes after electrophoresis, and were then blocked for 1 h at room temperature with 5% bovine serum albumin in TBST buffer. PVDF membranes were incubated overnight with an anti-GST antibody (CW0084, Beijing CWBio Co., Ltd., China). The PVDF membrane was subsequently hybridized with secondary horseradish peroxidase-conjugated goat anti-mouse IgG (CW0084, Beijing CWBio Co., Ltd., China) and the recombinant protein was detected by incubating with a chemiluminescence solution purchased from Thermo Scientific (#34079, Thermo Scientific Inc., Bremen, Germany) according to the manufacturer’s instructions.

### Lymphocyte separation, cell culture and proliferation assay

Five milliliters of whole blood was donated by a healthy volunteer. Lymphocytes were separated from non-coagulated whole blood using a human lymphocyte separation medium according to the manufacturer’s instructions (PAN Biotech, Aidenbach, Germany). The isolated cells were suspended in a culture bottle with high-glucose RPMI1640 medium (Gibco, UK) supplemented with 10% fetal bovine serum (Gibco, UK) and maintained at 37°C in a humidified 5% CO_2_ atmosphere overnight. After disposal of adherent cells, the suspended cells were recovered and treated with 5 μg ml^-1^ phytohemagglutinin for 72 h to expand the T cell population. For the T cell proliferation assay, cells were passaged and seeded in triplicate at 1 × 10^4^ cells per well in 96-well culture plates, followed by incubation with the corresponding treatment for another 72 h. The cell proliferation was analyzed by MTT assay. Cells treated with 5 μg ml^-1^ phytohemagglutinin served as the positive control. T-cell surface glycoprotein CD3 antibody (500 ng ml^-1^) was used to provide the first signal for T cell activation.

### Statistical analysis

All results were expressed as mean ± standard deviation of experiments performed in triplicate. Statistical differences were evaluated using a one-way analysis of variance with a Student’s *t*-test. Differences were considered to be statistically significant when P < 0.05.

## Competing interests

The authors declare that they have no competing interests.

## Authors’ contributions

XYL designed and conducted all experiments and prepared the manuscript. QQL participated in the construction of plasmids, expression and purification of recombinant protein. MCL prepared the polymer nanoparticles and participated in the immobilization of recombinant protein. YLZ participated in the design of cell purification and cultivation experiments and manuscript preparation. LLW carried out the cell culture and proliferation assay. All authors read and approved the final manuscript.
